# Leveraging Social Media to Promote Evidence-Based Continuing Medical Education

**DOI:** 10.1371/journal.pone.0168962

**Published:** 2017-01-06

**Authors:** Simone Flynn, Paul Hebert, Deborah Korenstein, Mark Ryan, William B. Jordan, Salomeh Keyhani

**Affiliations:** 1 National Physicians Alliance, Washington, DC, United States of America; 2 University of Washington School of Public Health, Department of Health Services, Seattle, Washington, United States of America; 3 Memorial Sloan Kettering Cancer Center, New York, New York, United States of America; 4 Virginia Commonwealth University, Department of Family Medicine and Population Health, Richmond, Virginia, United States of America; 5 Albert Einstein College of Medicine, Department of Family and Social Medicine, Bronx, New York, United States of America; 6 University of California, San Francisco, Division of Internal Medicine and the San Francisco Virginia, San Francisco, California, United States of America; University of Liverpool, UNITED KINGDOM

## Abstract

**Importance:**

New dissemination methods are needed to engage physicians in evidence-based continuing medical education (CME).

**Objective:**

To examine the effectiveness of social media in engaging physicians in non-industry-sponsored CME.

**Design:**

We tested the effect of different media platforms (e-mail, Facebook, paid Facebook and Twitter), CME topics, and different “hooks” (e.g., Q&A, clinical pearl and best evidence) on driving clicks to a landing site featuring non-industry sponsored CME. We modelled the effects of social media platform, CME topic, and hook using negative binomial regression on clicks to a single landing site. We used clicks to landing site adjusted for exposure and message number to calculate rate ratios. To understand how physicians interact with CME content on social media, we also conducted interviews with 10 physicians.

**Setting:**

The National Physicians Alliance (NPA) membership.

**Participants:**

NPA e-mail recipients, Facebook followers and friends, and Twitter followers.

**Main Outcomes and Measures:**

Clicks to the NPA’s CME landing site.

**Results:**

On average, 4,544 recipients received each message. Messages generated a total of 592 clicks to the landing site, for a rate of 5.4 clicks per 1000 recipients exposed. There were 5.4 clicks from e-mail, 11.9 clicks from Facebook, 5.5 clicks from paid Facebook, and 6.9 clicks from Twitter to the landing site for 1000 physicians exposed to each of 4 selected CME modules. A Facebook post generated 2.3x as many clicks to the landing site as did an e-mail after controlling for participant exposure, hook type and CME topic (p<0.001). Twitter posts (p = 0.13) and paid Facebook posts (p = 0.06) were not statistically different from e-mail in generating clicks to the landing site. Use of different hooks to engage physicians had no impact on clicks to the landing site. Interviews with physicians suggested that social media might not be a preferred vehicle for disseminating CME.

**Conclusions:**

Social media has a modest impact on driving traffic to evidence-based CME options. Facebook had a superior effect on driving physician web traffic to evidence-based CME compared to other social media platforms and email.

## Introduction

Pharmaceutical and medical-device marketing to physicians and patients is a steadily expanding, multi-billion dollar industry in the United States [1]. Continuing medical education (CME) is also a major recipient of commercial support. Industry-sponsored CME and its marketing has not only proliferated, but has come to dominate available CME offerings. Between 1998 and 2007, the share of CME provider income represented by commercial sources—and not including advertising and exhibits—grew from 34 to nearly 50 percent [2]. In 2014, the pharmaceutical and medical device industries provided $676 million or 25 percent of all CME funding in the United States [3]. Industry marketing materials generally influence provider prescribing practices [4] in terms of both quality and cost [5] and industry sponsorship of CME has been of particular concern. Both regulators and clinicians suspect bias in these educational materials [6–7] and there have been widespread calls for removal of industry from CME development [8].

In the past decade, academic detailing of physicians has been used as a strategic tool to combat the pharmaceutical industry’s influence on clinician prescribing habits by teaching about and encouraging evidence-based prescribing [9–10]. Rising use of social media may offer another opportunity to detail physicians with non-industry-sponsored, evidence-based CME, as an estimated 70 percent of physicians report using social media professionally [11].

Given the trends in use of social media by physicians, we examined whether social media can be useful in promoting CME to physicians. We specifically compared the reach of Facebook and Twitter to e-mail in engaging physicians in Agency for Healthcare Research & Quality (AHRQ) sponsored CME. We hypothesized that Twitter–a social media platform premised on brevity of message and ease of forwarding–would be most effective at directing physicians to CME coursework. We also examined whether certain types of messaging formats to engage physicians (“hook”) would be more effective compared to others (e.g. question and answer (Q&A) format vs. clinical pearl).

## Methods

### Overview

The research project was funded by an AHRQ research demonstration and dissemination grant and sought to test social media outreach efforts for informing physician members of medical organizations including professional societies about AHRQ’s Effective Health Care (EHC) CME resources.

We tested the effectiveness of using different social media platforms to direct members of the National Physicians Alliance (NPA) to AHRQ-developed CME. The NPA membership includes a multispecialty physician audience. The analytic design for this study consisted of three elements: CME topics, media platforms (e-mail, Facebook, paid Facebook, and Twitter), and hooks (Q&A, clinical pearl, best evidence, and control). By experimentally manipulating the combination and timing of CME topics, media platforms, and hooks in a Graeco-Latin Squares experimental design we disentangled the effects of each on directing physicians to CME. Finally, to better understand our findings we conducted interviews with ten NPA members who use social media.

### Study population

The sample included the NPA’s Facebook followers and friends, Twitter followers, and e-mail recipients who had opened at least one e-mail from the NPA in the prior 12 months. All subjects were members of the NPA or their professional contacts who voluntarily accessed our website. At the beginning of the data collection period, NPA had 5,245 e-mail recipients, 2,468 Facebook followers and 1,762 Twitter followers.

### Main outcome variable

The primary study outcome was clicks to the landing site. We created a landing site on the NPA website where visitors could click on links to AHRQ’s CME coursework. AHRQ-targeted links were maintained on the page throughout the study period, with the newest links spotlighted for easy access. A brief introduction on the landing page explained what happened when each of these links was clicked, and how to complete an AHRQ module and CME quiz.

The custom landing site served as the common address to which all social media efforts drove traffic. This arrangement enabled effective source tracking of incoming visitors to the page as well as monitoring outgoing traffic to AHRQ content. Our goal was to determine whether we could engage the target audience to click through to the landing site. Actual review of the AHRQ content was not the goal of study as we did not have user information and had no means of tracking who completed the CME content.

### Variables

#### CME Topics

The study team chose four AHRQ CME modules out of approximately 80 available at the time for use in the study. These four modules were chosen to represent a variety of topics that would be relevant to physicians with different specialty backgrounds:

1. No News May Not Be Good News: This CME module describes lapses in ambulatory test result follow-up and promotes a system-based solution [12].
2. E-Prescribing: E for Error?: This CME module defines the cost savings of e-prescribing and offers steps to limit errors associated with e-prescribing [13].
3. Outpatient Case Management for Adults with Medical Illness and Complex Care Needs: This CME module summarizes the effectiveness of case management programs, for patients with chronic and complex care needs, in improving clinical outcomes, quality of care, and patient/caregiver satisfaction [14].
4. Screening for Hepatitis C Virus Infection: This CME module reviews the clinical benefits, harms and limitations of screening for hepatitis C virus (HCV) infection in asymptomatic adults [15].

#### Media platforms

The media platforms consisted of e-mail, Facebook, paid Facebook, and Twitter. We made the decision to add a paid comparator to our study when Facebook unexpectedly changed their algorithm for organic reach (the number of Facebook followers that can see a particular post), thus limiting our ability to ensure that all our Facebook followers saw our message content. By using paid Facebook, all of our Facebook members could see the NPA posts that promoted CME modules. Paid Twitter was not available when this project was initiated.

#### Hooks

There were four different types of hooks: patient Q&A hook, clinical pearl hook, best evidence hook, and a control hook which provided a simple statement of CME topic. Hooks included posting a patient-care focused question about a CME-related topic, then linking to the answer and the CME content (patient Q&A hook); posting a tidbit of medical information or guidance related to the CME topic intended to improve a physician’s clinical practice, then linking to the CME content (clinical pearl hook); posting a link to research-based findings supporting a certain medical practice or approach related to the CME topic, along with a link to the CME content (best evidence hook); and posting a simple message simple message promoting the CME module without any of the other three communication strategies (control).

The purpose of the hook was to encourage physicians to engage with the targeted content. See Table A in S1 Appendix for an example of the four hook types used in this study.

### Intervention

We tested the effect of different CME topics, different media platforms (e-mail, Facebook, paid Facebook, and Twitter), and hooks on clicks to the NPA CME landing site. The goal of the study was to isolate the independent effects of the three factors of CME topic, media platform, and hook on the number of clicks to the landing site during a media campaign. To do this we used a block randomized design known as a Graeco-Latin Square. Each of the four “levels” [16] of media platform was paired, once with each “level” of CME topic and once with each hook, such that each factor was balanced with respect to the others. Each factor occurred 4 times over the course of the study, in a different combination each time. For example, in week one, the experiment was an e-mail containing the “No news not good news” CME topic with the control hook. The second use of e-mail came four months later when it was paired with the “E-prescribing” CME topic and the Q&A hook. In total, there were four experiments that used e-mail as the media platform, and e-mail was used once with each type of hook and message (Fig B in S1 Appendix).

**Fig 1:**
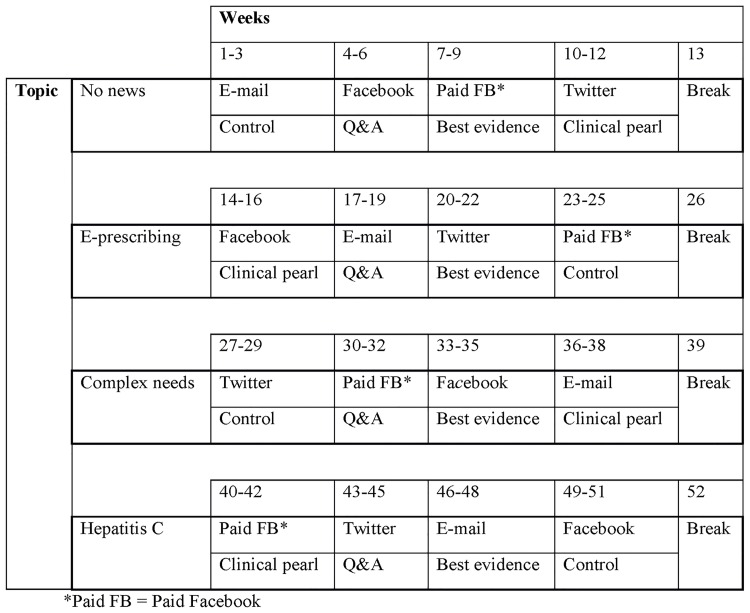
Intervention Schedule.

Each CME topic was also used with each media platform. For example, the CME module “E-Prescribing: E for Error?” was promoted with a different hook on each of our four different media platforms. The e-mail intervention involved sending six “Q&A” messages in total about the “E-Prescribing: E for Error?” CME module over three weeks, with protections built in to prevent supporters who opened a message from receiving a subsequent message in the same week. The Facebook intervention with the “E-Prescribing: E for Error?” CME module involved publishing a total of nine “clinical pearl” posts over a three-week period (Fig C in S1 Appendix). The paid Facebook intervention with the same “E-Prescribing: E for Error?” CME module involved publishing a total of nine “control” posts over a three-week period. The Twitter intervention involved nine “best evidence” tweets and an interactive tweet chat over a three-week period about the “E-Prescribing: E for Error?” CME module (Fig D in S1 Appendix). We limited e-mail messages to 6 rather than the 9 used for social media, because e-mail messages may be viewed more frequently by busy clinicians and we wanted to avoid e-mail subscription cancellations. Fig 1 displays the intervention schedule.

In summary, each media platform was used in four experiments each, resulting in a total of 16 experiments. Each experiment lasted 17 days for experiments using e-mail and 19 days for experiments using other media. The two-day difference in intervention length between e-mail and other media resulted from a Tuesday/Thursday send schedule for e-mail messages versus a Tuesday/Thursday/Saturday posting schedule for the other media platforms. There were between 3 and 12 days between experiments.

### Statistical analysis

The unit of analysis was the day on which a promotional campaign was conducted. Although the levels of the factors were balanced experimentally, the number of subjects exposed to each intervention varied substantially across media platform and within each media platform over time. To address this, we modeled the effects of CME topic, media platform, and hook using negative binomial regression, which is appropriate when the outcome is the count of events, like clicks to the landing site. To account for the clustering of observations across days within an experiment we used generalized estimating equations. We specified a log link, a negative binomial family, an independent correlation structure, and robust standard errors. We included indicator variables for each level of the three factors. Because 6 messages were sent per e-mail campaign whereas 9 messages were sent with other media, we also controlled for the message count. We controlled for exposure using the log of the number of recipients of each message on each day. Stata 13.1 was used for all analyses [17].

We also presented the results in terms of total clicks to the landing site for a campaign in which the number of recipients and messages was standardized. We used the results of the negative binomial model to estimate the effects on clicks to the landing site of a campaign in which each combination of CME topic, media platform, and hook was delivered, as 6 messages sent to 5000 recipients. We took this step only for purposes of presentation; it allowed us to present the results of the experiment in terms of clicks to the landing site, rather than the relative rates of clicks to the landing site that were the output of the negative binomial models. This step had no additional implications for inferences regarding the relative effectiveness of various CME topics, media, or hooks.

### Interviews

To better understand how physicians interact with CME content available on social media, we conducted interviews with ten practicing NPA-member physicians who use social media. We solicited physician participants by sending out an e-mail invitation to all physicians on the NPA e-mail list. Participation was incentivized through the offer of a $50 gift card to physicians who successfully completed their 30-minute phone interview. From the 26 physician respondents, we selected 10 physician participants who represented a wide-range of practice settings, medical specialties, ages, and years in practice. The interviews were conducted during a one-month period. The interview script included both open and closed-end questions to elicit information from the respondents about the relationship between CME and social media use (See Table E in S1 Appendix for a list of interview questions.) The phone interviews were recorded and transcribed and then analyzed by multiple members of the research team. Responses of shared opinion or subject matter were coded and grouped as a preliminary step in theme extraction.

Common themes were extracted when the answers of two or more respondents overlapped in opinion or subject matter. The themes were honed and finalized through multiple rounds of coding and discussion by researchers often referring back to the respondents’ specific word choice and range of possible meanings. For example, the research team discussed the different interpretations of the data that could be made with respondents’ use of the term “professional medical organization” vs. “medical-specialty society.” Due to the small size of the interview sample and complexity of responses, the project utilized human analyses by multiple researchers rather than qualitative coding software to extract themes.

## Results

### Clicks to the landing site across different social media platforms

There were four e-mail campaigns with 6 messages each for a total of 24 messages (Table 1). On average, 4,544 recipients received each message, and these messages generated a total of 592 clicks to the landing site, for a rate of 5.4 clicks per 1000 recipients exposed (i.e., 5.4 = 1000*592/(24*4544)). There were an average of 11.9 clicks from Facebook, 5.5 clicks from paid Facebook, and 6.9 clicks from Twitter to the landing site per 1000 physicians exposed to a message. Similar figures are shown for each variable of CME topic, media platform, and hook. In unadjusted analyses, e-mail and paid Facebook generated more clicks to the landing site, although this was largely due to the far greater number of recipients exposed to the messages.

**Table 1:** Total clicks to the landing site.

	Messages sent	Total clicks to the landing site	Subjects exposed per message, mean (sd)	Clicks per 1000 subjects exposed, mean(se)
**CME Topic**				
No news not be good news	33	421	1688.0 (2045)	7.6 (0.9)
E-prescribing	33	235	1273.9 (1674)	5.6 (0.7)
Outpatient case management	33	295	1349.6 (1668)	6.6 (0.7)
Screening for Hepatitis c	33	142	1594.9 (1913.4)	2.7 (0.4)
**Media Platform**				
E-mail	24	592	4544.3 (546.0)	5.4 (0.7)
Facebook	36	43	100.4 (62.2)	11.9 (2.4)
Paid Facebook	36	422	2139.3 (1227)	5.5 (0.5)
Twitter	36	36	145.0 (76.3)	6.9 (1.2)
**Hook**				
Q&A	33	236	1344.5 (1681)	5.3 (0.6)
Clinical pearl	33	275	1575.6 (1901)	5.3 (0.6)
Best evidence	33	210	1673.0 (2025)	3.8 (0.6)
Control	33	372	1313.4 (1706)	8.6 (0.6)

### Clicks to landing site after adjusting for exposure and message number

Table 2 shows the results of the negative binomial regression. Rate ratios and associated p-values are presented. The rate ratio for a given level of a factor is the adjusted clicks to the landing site per day for that level relative to reference level of the factor. For example, a rate ratio of 2.34 for Facebook indicates that adjusting for exposure and message count, a Facebook post generates 2.3x as many clicks to the landing site as does an e-mail message. Facebook was the only media platform that was statistically significantly different from e-mail messaging. The CME topics of “Outpatient Case Management” and “Screening for Hep C” generated statistically significantly lower clicks to the landing site than did “No News May Not Be Good News.” There were not statistically significant differences in the effects of the hook on clicks to the landing site. The number of clicks to the landing site generally declined with each subsequent message within a campaign.

**Table 2:** Rate ratios for clicks to landing site, by CME topic, media platform, and hook, adjusted for exposure and message number.

	Rate Ratio	p-value
CME Topic (Module)		
M1. No news not good news	1.0 (reference)	
M2. E-prescribing	0.78	(0.238)
M3. Outpatient case management	0.70*	(0.020)
M4. Screening for Hep C	0.37***	(0.000)
Media Platform		
e-mail	1.0 (reference)	
Facebook	2.34***	(0.000)
Paid Facebook	1.31	(0.064)
Twitter	1.43	(0.134)
Hook		
A. Q&A	0.91	(0.418)
B. Clinical Pearl	0.87	(0.370)
C. Best Evidence	0.74	(0.119)
D. Control	1.0 (reference)	
Message number		
1	1.0 (reference)	
2	0.78	(0.262)
3	0.54**	(0.007)
4	0.54***	(0.000)
5	0.55**	(0.010)
6	0.47**	(0.009)
7	0.71	(0.273)
8	0.63	(0.214)
9	0.48**	(0.005)
Observations	132	

Exponentiated coefficients; *p*-values in parentheses

* *p* < 0.05,

** *p* < 0.01,

*** *p* < 0.001

**Fig 2:**
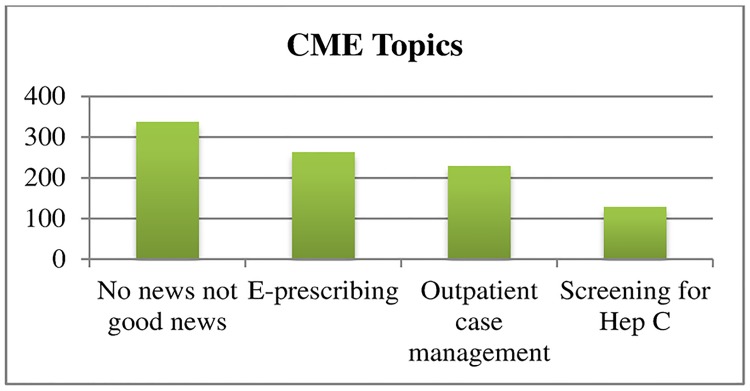
Estimated number of clicks by CME topic to landing site for a hypothetical campaign of six messages sent to 5000 recipients.

**Fig 3:**
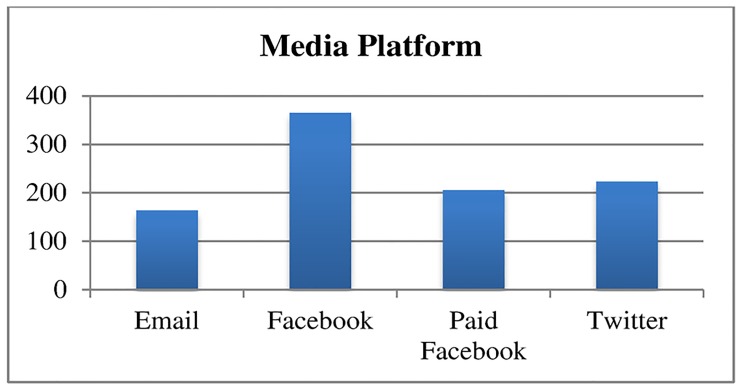
Estimated number of clicks by social media platform to landing site for a hypothetical campaign of six messages sent to 5000 recipients.

**Fig 4:**
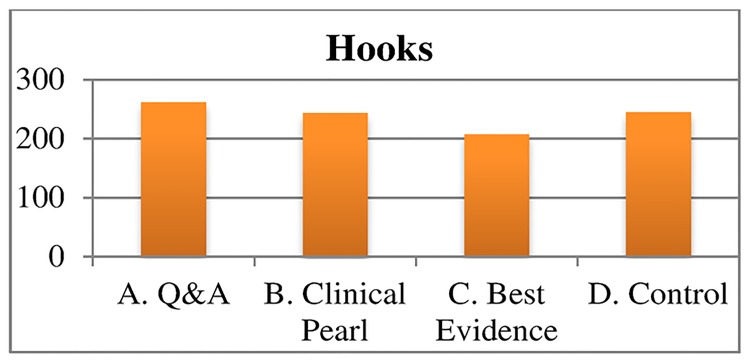
Estimated number of clicks by hooks to landing site for a hypothetical campaign of six messages sent to 5000 recipients.

To standardize the exposure and visually demonstrate the effect of media platform on clicks to the landing site, we also examined the clicks to the landing site for a hypothetical campaign that includes 6 message blasts to 5,000 recipients. The 5,000-recipient model assumes that the e-mail, Facebook, paid Facebook, and Twitter samples each had a level of engagement and interest in CME equal to the sample from the study (Figs 2–4). The figure illustrates that Facebook was more successful at engaging physicians to click through to the landing site. It also illustrates the lower rate of enthusiasm for some CME topics compared to others and the lack of impact of different hooks on click-through rates.

### Interviews

We extracted 7 themes that described physicians’ views regarding the roles of social media in CME dissemination: 1) Reliance on established sources and pathways of CME (colleague recommendations and medical specialty society CME offerings); 2) Abundance of CME offered in physicians’ professional environments; 3) Mismatch between transience of social media, and planned completion of durable CME materials; 4) Physicians’ career and/or personal life time pressures leaving little time for online CME; 5) Association of particular social media platforms with either personal or career spheres; 6) Physicians’ concern for their privacy online; and 7) Seasonal and/or time-specific nature of need for CME. In addition, participants expressed the belief that pairing with medical specialty societies may be the most effective way to promote a no-cost, industry-free CME option to physicians. To better illustrate these themes, we have included quotes identified with each theme in Table 3 (a complete version is provided in the S1 Appendix).

**Table 3:** Example Quotes from Interviews with Physicians.

Themes	Physician Quotes
1) Physicians rely on established sources and pathways of CME (colleague recommendations and professional medical specialty society CME offerings)	“I primarily look for CME from my own organization AAPMR, the American Academy of Physical Medicine and Rehabilitation, because they’re the ones that are going to count the most and be the most applicable.”
2) Abundance of CME offered in physicians’ professional environments	“I don’t actively look for CME credit probably because I work at an academic institution. I already get a lot of CME as I go to a lot of national conferences. I fulfill that need in my professional development very easily.”
3) Mismatch between transience of social media, and planned completion of durable CME materials	“I think of a lot of social media as a major time suck and I feel like it’s a no overall because it [social media] is incredibly transient and not consistent. I mean I like that I know that on the American Academy of Pediatrics website I will be able to find it when I to get to it whereas most of social media I feel is so transient I feel like wouldn’t know what to rely on if I wanted to find something consistently.”
4) Physicians’ career/life time pressures leave little time for online CME	“I haven't completed online CME and it’s because I generally have a lot of things to do in my life related to my job(s) plural and I’m not able to really discipline myself enough to sit down and do something online for which there’s not a crunch or a clear outcome that I’m seeking.”
5) Association of different social media platforms with personal and career spheres	“I don’t use Twitter. I use other things. Mainly I don’t see a purpose to it. I use Facebook to connect with my friends, my current life, my past life, high school, college, etc., and to keep track of people. I know people use it for more business and marketing purposes but I think it’s difficult for medicine other than just posting articles and updating people on events that you’re covering. I use LinkedIn to connect with other professionals in the medical profession. “
6) Physicians’ concern for their privacy online	“I keep a pretty low profile on Facebook because I’m aware of its quasi-public nature and I don’t use it as a platform I should say. I use it almost as an information source for myself but not generally to spread my own messages.”
7) CME is seasonal for many physicians	“I’m certainly aware of the national conferences that are pertinent to my specialty so I kind of put that into my schedule a year in advance.”

## Discussion

Overall the impact of social media was modest and most forms of social media were similar to email in driving physicians to non-industry sponsored CME. Facebook was the only form of social media that outperformed e-mail in driving traffic to evidence-based CME content. As e-mail has become a standard media outlet for advertising CME, the favorable relative performance of Facebook shows promise.

The non-profit industry reports an average engagement rate of 5.4% for Facebook and 1.6% for Twitter, with this figure extending beyond click through rates to include comments, shares, retweets, and likes [18]. When isolating for click through rates, recent medical internet research studies have yielded social media click through rates ranging from 0.2% overall for paid promotion of maternity care quality information across three social media platforms [19]; to 0.1% overall for paid promotion to recruit Latino smokers to an internet cessation program on four social media platforms [20]; and 0.03% for paid promotion of clinical practice guidelines on Facebook [21]. The variability in social media click-through rates for clinician and patient healthcare campaigns suggests that we are in the early stages of establishing social media benchmarks, but our rates compared favorably with what is known from the literature.

Our data did not confirm the project’s overall hypothesis that Twitter would be most effective at engaging our physician audience in CME coursework. Factors affecting the life cycle and longevity of tweets, such as retweeting and activity level of follower Twitter streams, may have influenced our Twitter results [22]. Finally, during the period of the study Twitter was based on strict chronology, which may provide a less targeted promotional platform. Since completion of our study, Twitter has moved from strict chronology to an algorithmic structure, which may impact its performance in future similar studies [23].

Facebook was the only media platform that was statistically significantly different from e-mail messaging, with a Facebook post generating 2.3x as many clicks to the landing site as does an e-mail message. Our finding that Facebook posts produced more clicks to the landing site than e-mail or Twitter might be explained by multiple factors. Rather than provide a stream of content from social connections in plain chronological order, Facebook’s algorithm sorts and ranks content to show users content that Facebook has determined will be of the most interest [24]. It is reasonable to assume that, based on how Facebook’s algorithm is purported to work, Facebook showed our CME posts to followers with the highest level of interest in some facet of the CME topic [25]. Paid promotion on Facebook on the other hand generated lower click-through rates than posts to NPA’s Facebook followers. It may be that the algorithm of posts from social connections on Facebook is better at targeting content than the algorithm for paid advertisements (ads). The discrepancy in click-through rates on Facebook and paid Facebook may also be due to Facebook users reacting differently to paid content on their Facebook news feeds.

Enthusiasm varied across CME content. The CME topics of “Outpatient Case Management” and “Screening for Hep C” generated statistically significantly lower clicks to the landing site than did “No News May Not Be Good News.” This was not surprising. The NPA has a multispecialty membership; outpatient case management and screening for hepatitis C may be more of interest to primary care providers than specialists, while the need for appropriate follow up of test results is more broadly applicable. The number of clicks to the landing site generally declined with each subsequent message within a campaign. This finding suggests that social media followers may have reached saturation with different messages before our intervention concluded. Continued exposure did not create increased interest or engagement.

While Facebook compared favorably to email in driving clicks to the landing sites, in absolute terms the impact of social media was modest and most forms of social media were similar to email. Perhaps social media may be more successful for scientific dissemination to the public. For example, research *has* demonstrated the positive potential of social media in patient education and patient health choices. In a study that evaluated an evidence-based social-networking intervention aimed at reducing Chlamydia among adolescents, 81% of subjects reported that the study’s intervention of STD education disseminated through Facebook influenced their decision to practice safe sex in future sexual experiences [26]. Other researchers have found that public health education through a website for hepatitis B education–with topic pages on daily health care, symptoms and route of infection–improves participant knowledge and awareness of risk factors for hepatitis B infection [27]. Research has also demonstrated that Twitter can effectively be used to increase the reach and dissemination of conference research findings [28].

However, findings of studies that evaluate the impact of social media dissemination on physician uptake of clinical resources are less encouraging. For example, studies of the impact of social media on physician awareness of clinical practice guidelines (CPG) found that social media-based dissemination methods did not improve CPG awareness compared to traditional print, e-mail, or internet-based methods. The investigators in this study speculated that research on message delivery will be required to improve guideline uptake [29]. In contrast to this earlier work, our intervention included variations in message delivery using different hooks (Q&A, clinical pearl, best evidence, and control), but varying these formats did not impact uptake of CME content.

One hypothesis for the overall limited impact of social media in engaging physicians in clinical practice guideline awareness or CME may be that physicians have not traditionally looked to social media for professional development. Our interviews with 10 NPA members who use social media suggested that social media might not be a preferred vehicle for disseminating medical content and that physicians may instead still turn mainly to their own medical specialty society or place of employment for CME opportunities. To effectively promote CME, it is unlikely that any single media platform will achieve satisfying reach in the next few years, given the dominance of established pathways for CME promotion and physicians’ still widely varying engagement with social media. If physician behavior follows demographic trends in the general population, it is possible that the influence of social media will evolve over time, and CME promotion strategies will involve simultaneous use of traditional and newer media outlets in a fragmented media landscape.

### Limitations of study

This study has limitations that deserve comment. While the research team endeavored to choose AHRQ CME topics that were appealing to a wide range of physician specialties, the reality of specialty and employer-specific CME requirements for physicians likely influenced physician interest in and uptake of the CME topics promoted in this study. In addition, we tested the effectiveness of social media on driving traffic to evidence-based CME content, not the actual use of this content. However, it is likely that content uptake is impacted by the route by which users access it. The lack of scalability and control inherent in our promotion and dissemination efforts on Facebook was also a significant limitation in our evidence-based CME dissemination efforts. During the study period, Facebook changed the “organic reach” algorithm they used to determine which and how many of an organization’s Facebook followers saw that organization’s Facebook posts. This unanticipated change directly influenced our ability to depend on consistent numeric denominators in our analysis of Facebook reach, prompting us to add a “paid” Facebook arm to the study. Finally, given the relatively small scale of exposure and response rates, we were unable analyze clicks to the AHRQ CME page as the main outcome variable. Future studies with larger sample sizes are needed for such an analysis.

Generalizing some of our findings to all physicians may also be challenging. Specific differences in follower demographics between NPA’s Facebook followers and friends, Twitter followers, and e-mail recipients are not known but general differences between individual followers across social media platforms likely include different level of engagement with the NPA organization, donation history, type of healthcare career and/or specialty, and niche healthcare-related interests. While NPA members and social media followers form a loose and dispersed community of practice, further targeted research would be needed to track the how the presence or absence of well-defined communities of practice and nodes of influence around key social media users in the health professions influences uptake of industry-free CME resources. Since its inception, the NPA has refused all funding from pharmaceutical and medical device companies and worked to reduce such conflicts of interest within the medical profession; the organization’s affiliated physicians may be more enthusiastic than the average physician about using non-industry sponsored CME. Members of the NPA may differ with the general physician population in their enthusiasm in use of social media for the purpose of education. Lastly, the length and design of the study–with NPA followers being exposed to AHRQ CME content repeatedly across social media over a 12-month period—may have created response fatigue in our sample.

### Conclusions

Social media overall has a modest impact on driving traffic to evidence-based CME options. Among the different modalities tested, Facebook led to the highest click through rate. While the algorithm developed and instituted by Facebook places limits on the ability of any physician organization to specifically target its members, the algorithm is slightly better than email at reaching an audience.

## Supporting Information

S1 Appendix (DOCX)

## References

[1] Primack B. Media literacy to improve evidence-based prescribing among family medicine trainees. National Association for Media Literacy Education, Conference, 2011.

[2] Lo B, Field MJ U.S. Committee on Conflict of Interest in Medical Research, Education, and Practice, eds. *Conflict of Interest in Medical Research*, *Education*, *and Practice*. Institute of Medicine. National Academies Press; 2009. 20662118

[3] Accreditation Council for Continuing Medical Education (ACCME) 2014 Annual Report. Accreditation Council for Continuing Medical Education. http://www.accme.org/sites/default/files/630_20150707_2014_Annual_Report.pdf. Accessed April 5, 2016.

[4] Steinman MA, Landefeld CS, Baron RB. Industry support of CME: are we at the tipping point? *New England Journal of Medicine*. 2012;366(12):1069–71. 10.1056/NEJMp1114776 22435367

[5] Spurling GK, Mansfield PR, Montgomery BD, et al Information from Pharmaceutical Companies and the Quality, Quantity, and Cost of Physicians’ Prescribing: A Systematic Review. *PLOS Medicine*. 2010;7(10):e1000352 Accessed April 26, 2016. 10.1371/journal.pmed.1000352 20976098 PMC2957394

[6] Spithoff S. Industry involvement in continuing medical education: Time to say no. Canadian Family Physician. 2014;60:8694–696. http://www.cfp.ca/content/60/8/694.long. Accessed April 26, 2016. PMC4131951 25122806

[7] Tabas JA, Boscardin C, Jacobsen DM, Steinman MA, Volberding PA, Baron RB. Clinician attitudes about commercial support of continuing medical education: results of a detailed survey. *Arch Intern Med*. 2011;171(9):840–6. Accessed April 26, 2016. 10.1001/archinternmed.2011.179 21555662

[8] Steinman MA, Landefeld CS, Baron RB. Industry support of CME: are we at the tipping point? *New England Journal of Medicine*. 2012;366(12):1069–71. 10.1056/NEJMp1114776 22435367

[9] Academic Detailing for Healthcare Professionals. Center for Disease Control and Prevention. http://www.cdc.gov/getsmart/community/improving-prescribing/interventions/academic-detailing.html. Accessed April 5, 2016.

[10] Spithoff S. Industry involvement in continuing medical education: Time to say no. *Canadian Family Physician*. 2014;60:8694–696. http://www.cfp.ca/content/60/8/694.long. Accessed April 26, 2016. PMC4131951 25122806

[11] McGowan BS, Wasko M, Vartabedian BS, Miller RS, Freiherr DD, Abdolrasulnia M. Understanding the Factors That Influence the Adoption and Meaningful Use of Social Media by Physicians to Share Medical Information. J Med Internet Res. 2012;14(5):e117 Accessed April 25, 2016. 10.2196/jmir.2138 23006336 PMC3510763

[12] No News May Not Be Good News. Agency for Healthcare Research and Quality. http://webmm.ahrq.gov/case.aspx?caseID=275. Accessed April 5, 2016.

[13] E-Prescribing: E for Error? Agency for Healthcare Research and Quality. https://psnet.ahrq.gov/webmm/case/260. Accessed April 5, 2016.

[14] Outpatient Case Management for Adults With Medical Illness and Complex Care Needs. Agency for Healthcare Research and Quality. http://www.baylorcme.org/trans/cme.cfm?activityID=349. Accessed April 5, 2016. 23346604

[15] Screening for Hepatitis C Virus Infection. Agency for Healthcare Research and Quality. http://www.baylorcme.org/trans/cme.cfm?activityID=353. Accessed April 5, 2016.

[16] Fisher RA. The design of Experiments, 8^th^ edition 1966;82–84.

[17] StataCorp. 2013 Stata Statistical Software: Release 13. College Station, TX: StataCorp LP.

[18] 2015 M+R Benchmarks Study. M+R. http://mrbenchmarks.com/#bys. Accessed May 20, 2016.

[19] Huesch M.D., Galstyan A., Ong M.K. and Doctor J.N. Using Social Media, Online Social Networks, and Internet Search as Platforms for Public Health Interventions: A Pilot Study. *Health Services Research*. 2016. Accessed May 20, 2016. 10.1111/1475-6773.12496 PMC4874940 27161093

[20] Graham AL, Fang Y, Moreno JL, et al Online Advertising to Reach and Recruit Latino Smokers to an Internet Cessation Program: Impact and Costs. *J Med Internet Res*. 2012;14(4):e116 Accessed May 20, 2016. 10.2196/jmir.2162 22954502 PMC3510691

[21] Narayanaswami P, Gronseth G, Dubinsky R, et al The Impact of Social Media on Dissemination and Implementation of Clinical Practice Guidelines: A Longitudinal Observational Study. *J Med Internet Res*. 2015;17(8):e193 Accessed May 20, 2016. 10.2196/jmir.4414 26272267 PMC4736287

[22] Willis A, Fisher A, Lvov I. Mapping networks of influence: tracking Twitter conversations through time and space. *Participations*: *Journal of Audience & Reception Studies*. 2015;12(1): 494–530. http://oro.open.ac.uk/43281/1/30.pdf. Accessed April 19, 2016.

[23] Wagner, K. Twitter Isn't Becoming Facebook, but It Is Using More Algorithms. RedCode. 2016. http://www.recode.net/2016/2/10/11587710/twitter-isnt-becoming-facebook-but-it-is-using-more-algorithms. Accessed May 14, 2016.

[24] Sullivan G. How Facebook and Twitter control what you see about Ferguson. Washington Post. 2014. https://www.washingtonpost.com/news/morning-mix/wp/2014/08/19/how-facebook-and-twitter-control-what-you-see-about-ferguson/. Accessed May 14, 2016.

[25] An Update to News Feed: What it Means for Businesses. Facebook. https://www.facebook.com/business/news/update-to-facebook-news-feed. Accessed April 10, 2016.

[26] Jones K, Baldwin KA, Lewis PR. The potential influence of a social media intervention on risky sexual behavior and Chlamydia incidence. *J Community Health Nurs*. 2012;29(2):106–120. http://www.ncbi.nlm.nih.gov/pubmed/22536914?dopt=Abstract. Accessed April 5, 2016. 10.1080/07370016.2012.670579 22536914

[27] Huang Y, Hung C. The effect of health education through the internet on university female students’ hepatitis B knowledge and cognition. Journal of Clinical Nursing. 2009;18: 3342–3348. Accessed April 15, 2016. 10.1111/j.1365-2702.2009.02907.x 19732240

[28] Ferguson C, Inglis SC, Newton PJ, et al Social media: a tool to spread information: a case study analysis of twitter conversation at the Cardiac Society of Australia & New Zealand 61st annual scientific meeting 2013. *Collegian*. 2014;21(2):89–93. http://www.ncbi.nlm.nih.gov/pubmed/25109206?dopt=Abstract. Accessed April 5, 2016. 25109206 10.1016/j.colegn.2014.03.002

[29] Narayanaswami P, Gronseth G, Dubinsky R, et al The Impact of Social Media on Dissemination and Implementation of Clinical Practice Guidelines: A Longitudinal Observational Study. *J Med Internet Res*. 2015;17(8):e193 Accessed April 5, 2016. 10.2196/jmir.4414 26272267 PMC4736287

