# Supplementary material for: Leveraging Social Media to Promote Evidence-Based Continuing Medical Education
Source: PLoS One. 2017 Jan 6;12(1):e0168962. doi: 10.1371/journal.pone.0168962 (PMC5218469; doi:10.1371/journal.pone.0168962)
Supplement: S1 Appendix — (DOCX) [file pone.0168962.s001.docx]

**S1 Appendix**

**Table A. Example of four hook types posted to Facebook.**

| Hook Type | Example posts |
| --- | --- |
| Patient Q&A | Question: What percentage of all medical errors occurring in ambulatory settings are due to the inadequate follow-up of abnormal test results? Answer: One-quarter. Learn more: "No News May Not Be Good News" AHRQ’s industry-free CME module [Landing page URL]. |
| Clinical Pearl | Maximize safe e-prescribing performance: When a prescription is canceled or changed, follow up with a phone call to the pharmacy. Learn more: "E-Prescribing: E for Error?" AHRQ’s industry-free CME module [Landing page URL]. |
| Best Evidence | Chronic diseases are leading cause of illness, disability + death in the US. Check out best evidence on chronic illness case management: “Outpatient Case Management for Adults W/ Medical Illness + Complex Care Needs” AHRQ’s industry-free CME [Landing page URL] and “Integrating education, group support, and case management for diabetic Hispanics” [Link]. |
| Control | Learn best practices for screening for HCV infection with AHRQ’s industry-free CME module, “Screening for Hepatitis C Virus Infection” [Landing page URL]. |

**Fig B. Example e-mail sent to NPA members during study.**


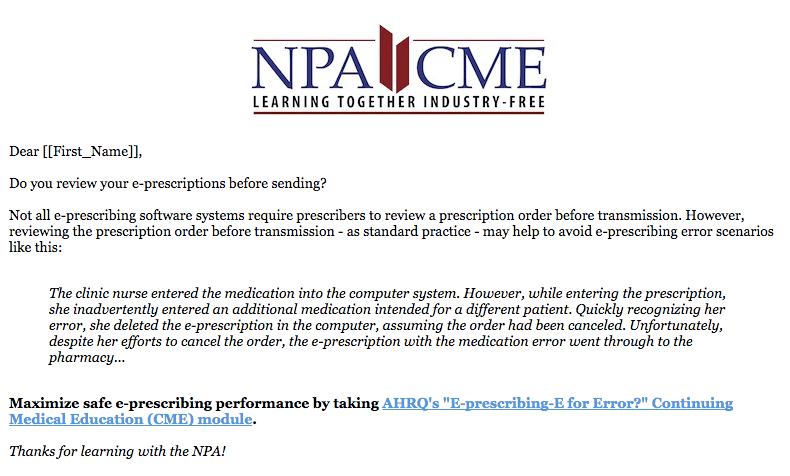


**Fig C. Example Facebook post published during the study.**


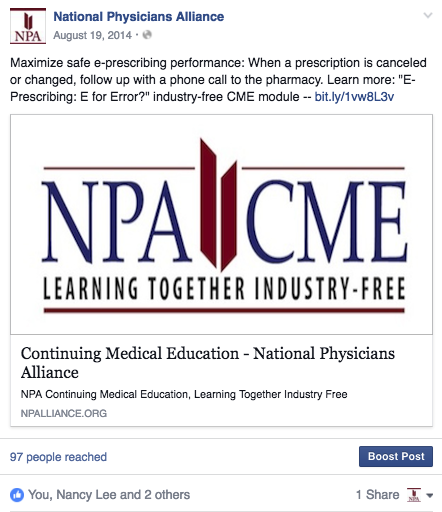


**Fig D. Example tweet published during the study.**


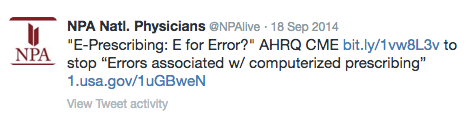


**Table E. AHRQ Interview Question Script.**

| Introduction | Thank you for agreeing to this interview. As part of a grant-funded  research project, NPA is interviewing practicing physicians who have finished residency and also use social media. Participants who  complete the interview will receive a $50 Amazon gift card. Please  note that this interview is being recorded for research purposes. |
| --- | --- |
| # 1 | For what purposes do you use Twitter? Do you use it to keep up with friends? Do you use it for political advocacy? Do you use it for  professional purposes? If yes, in what way? |
| *#2* | For what purposes do you use Facebook? Do you use it to keep up with friends? Do you use it for political advocacy? Do you use it for professional purposes? If yes, in what way? |
| *#3* | For what purposes do you use LinkedIn? Do you use it to keep up with friends? Do you use it for political advocacy? Do you use it for professional purposes? If yes, in what way? |
| *#4* | For what purposes do you use LinkedIn? Do you use it to keep up with friends? Do you use it for political advocacy? Do you use it for professional purposes? If yes, in what way? |
| *#5* | For what purposes do you use Google+? Do you use it to keep up with friends? Do you use it for political advocacy? Do you use it for professional purposes? If yes, in what way? |
| *#6* | Have you ever used social media for identifying CME opportunities? Why or why not? |
| *#7* | Have you ever become engaged in CME opportunities through social media? Why or why not? |
| *#8* | Do you think of social media as a good vehicle to identify CME? Why or why not? |
| *#9* | Which social medial platform, if any, do you think would be particularly useful to identify CME? |
| *#10* | Do you actively look for sources of CME credit? Why or why not? If yes, where do you look for CME resources? |
| *#11* | When in the calendar year or accreditation cycle do you look for CME? |
| *#12* | When you identify potential CME resources, do you look at the sponsor? If yes, what do you look for? |
| *#13* | Have you ever completed online CME? Why or why not? If yes, where did you learn about the online CME resource? Who sponsored/provided the CME? Was there a cost to complete and receive credit? |
| *#14* | How do you think a no-cost, industry-free CME option could most effectively be promoted to physicians? |
| *#15* | Were you aware that NPA has a grant to study social media dissemination  strategies for AHRQ CME modules? |
| *#16* | Have you participated in any NPA-promoted AHRQ CME modules within the  last year? Why or why not? |

**Table F. Example Quotes from Interviews with Physicians.**

| **Themes** | **Physician Quotes** |
| --- | --- |
| 1) Physicians rely on established sources and pathways of CME (colleague recommendations and professional medical specialty society CME offerings) | “I primarily look for CME from my own organization AAPMR, the American Academy of Physical Medicine and Rehabilitation, because they’re the ones that are going to count the most and be the most applicable.” |
|  | “I’m much more likely to look at it [CME opportunities] if my work people send the e-mail.” |
|  | “I don’t look for CME credit. It’s much more passive. It’s just whatever the department puts out and then whatever other physicians e-mail or forward on.” |
| 2) Abundance of CME offered in physicians’ professional environments | “Because I’m in an academic setting and I’m going to conferences, CME finds me for the most part.” |
|  | “I don’t actively look for CME credit probably because I work at an academic institution. I already get a lot of CME as I go to a lot of national conferences. I fulfill that need in my professional development very easily.” |
|  | “I don’t actively look for sources of CME credit because I have to attend a lot of professional meetings already so I don’t ever need CME and I do a lot of teaching so I also don’t need CME.” |
|  | “I’m involved in a lot of professional organizations that deliver CME so I’m in no need of more CME.” |
| 3) Mismatch between transience of social media, and planned completion of durable CME materials | “I think of a lot of social media as a major time suck and I feel like it’s a no overall because it [social media] is incredible transient and not a consistent. I mean I like that I know that on the American Academy of Pediatrics website I will be able to find it when I to get to it whereas most of social media I feel is so transient I feel like wouldn’t know what to rely on if I wanted to find something consistently.” |
|  | “You get information very quickly online and, at this point, I’ll get an e-mail [about CME opportunity] and save it and then come back to it but something on Twitter, I’ll see it and it’ll zoom by so it doesn’t have that same staying power for me to be organized enough to pursue something like that.” |
|  | “I don’t think social media is a good vehicle for promoting CME mainly because the people who are using social media are already aware of CME opportunities. You know? People who are already using social media are actively checking the websites of their professional organizations so I don’t think they’re being surprised by new CME opportunities. Most CME opportunities are pretty standard. They occur at the same times of the year. They’re offered by the same organizations. Um, so I don’t think are very many new, surprising CME opportunities that I’d only hear about on social media.” |
| 4) Physicians’ career/life time pressures leave little time for online CME | “I haven't completed online CME and it’s because I generally have a lot of things to do in my life related to my job(s) plural and I’m not able to really discipline myself enough to sit down and do something online for which there’s not a crunch or a clear outcome that I’m seeking.” |
| 5) Association of different social media platforms with personal and career spheres | “I don’t use Twitter. I use other things. Mainly I don’t see a purpose to it. I use Facebook to connect with my friends, my current life, my past life, high school, college, etc., and to keep track of people. I know people use it for more business and marketing purposes but I think it’s difficult for medicine other than just posting articles and updating people on events that you’re covering. I use LinkedIn to connect with other professionals in the medical profession.” |
|  | “I use Twitter for professional purposes, to follow news and sort of build by own brand. Facebook is mainly personal. I use to stay in touch with my friends.” |
|  | “I have a professional Twitter account. I don’t think patients really see it. It’s more for my colleagues that I meet in my department or at other institutions when I’m at conferences. I have a personal Facebook account but I’m using it less and less.” |
| 6) Physicians’ concern for their privacy online | “I have changed my name on Facebook so that patients wouldn’t be able to find me…well, they probably could if they wanted to but I’m somewhat protective of that and have a lot of securities in place so that it’s not a professional mode of social media for me.” |
|  | “I keep a pretty low profile on Facebook because I’m aware of its quasi-public nature and I don’t use it as a platform I should say. I use it almost as an information source for myself but not generally to spread my own messages.” |
| 7) CME is seasonal for many physicians | “I’m certainly aware of the national conferences that are pertinent to my specialty so I kind of put that into my schedule a year in advance.” |
|  | “I’m aware of when my meetings are with the Society for the Teachers of Family Medicine and Family Medicine Education Consortium so I just plan to go to those meetings.” |
| 8) Belief that pairing with medical specialty societies may be most effective way to promote a no-cost, industry-free CME option to physicians | “[Promote CME] through a trusted network. So for those who are already members of NPA, through that, or if NPA were offering it, they could network through other professional entities either the big ones like AMA, AAP, things like that.” |
|  | “If you’re looking to target, you know, a certain regional population or segment of the medical community then pairing up with local professional organizations, you know, the AMA, the AOA, state societies, county societies and asking them to promote to their members because they already have lists of people who are interested in attending those things. You’d have some partners to advertise for you.” |
